# Advanced specificity and sensitivity studies relative to a research use only transcription-mediated amplification-based assay for *Treponema pallidum* RNA detection

**DOI:** 10.1128/jcm.00388-25

**Published:** 2025-06-20

**Authors:** Trinity Krueger, Grace Kadonsky, Elizabeth Thelen, Amanda Zapp, Josephine Moore, Ahmet Muslu, Allan Pillay, Irene A. Stafford, Erik Munson

**Affiliations:** ^1^Department of Medical Laboratory Science, Marquette University 5505, Milwaukee, Wisconsin, USA; 2 Division of STD Prevention, Centers for Disease Control and Prevention 1242, Atlanta, Georgia, USA; 3 Department of Obstetrics and Gynecology, Division of Maternal Fetal Medicine, McGovern Medical School at UTHealth 12339 , Houston, Texas, USA; 4 Wisconsin Clinical Laboratory Network Laboratory Technical Advisory Group, Madison, Wisconsin, USA; Endeavor Health, Evanston, Illinois, USA

**Keywords:** rectal swab specimen, transcription-mediated amplification, *Treponema pallidum*

## Abstract

Preliminary experimentation has suggested that a research-use-only real-time transcription-mediated amplification assay for *Treponema pallidum* (RUO *T. pallidum* TMA) yields instances of *T. pallidum* nucleic acid detection that coincide with non-treponemal serology in men who have sex with men (MSM) at increased risk for sexually transmitted infection. To further characterize the specificity of RUO *T. pallidum* TMA testing, 3,586 rectal swab specimens reported as “not detected” by the assay generated a mean endpoint FAM fluorescence of 637.5 units. Introduction of *Treponema denticola*, *Treponema phagedenis*, and *Treponema refringens* nucleic acid into matrices generated mean endpoint FAM fluorescence ranging from 620.7 to 633.5 units (*P* ≥ 0.15 versus control). Introduction of lubricant to a pooled rectal swab matrix did not result in elevated FAM fluorescence (mean endpoint value 628.3 units [95% CI 612.0, 644.6]; *P* = 0.67). Introduction of talcum powder, urine, seminal fluid, and blood also failed to generate increased FAM fluorescence (*P* ≥ 0.20). Analytic sensitivity assessment was measured by serial 10-fold dilution of *T. pallidum* whole organism or *in vitro* 23S rRNA transcript in specimen transport medium or pooled rectal swab matrix and interrogation by the assay. Probit analysis estimated sensitivity (95% detection) of RUO *T. pallidum* TMA at 421–5,707 *in vitro* transcript copies/mL and 9–48 *T. pallidum* cells/mL, depending on dilution matrix. These results support RUO *T. pallidum* TMA as a highly sensitive method for *T. pallidum* detection that is not impacted by potentially cross-reactive organisms or interfering substances and may have adjunctive diagnosis capability for syphilis in MSM.

**IMPORTANCE:**

Research-use-only *Treponema pallidum* transcription-mediated amplification (RUO *T. pallidum* TMA) has the potential to improve laboratory diagnosis of syphilis, particularly in patients at increased risk for sexually transmitted infection. The high analytic sensitivity and lack of cross-reactivity of the assay can facilitate other laboratories exploring the use of the test in a research setting.

## INTRODUCTION

Surveys have revealed that primary and secondary syphilis rates among men who have sex with men (MSM) are 100 times higher than those among men who have sex with women (1). While combination treponemal and non-treponemal serology continues to be the laboratory diagnostic reference standard for evidence of *Treponema pallidum* infection, molecular diagnostics can contribute adjunctive or additive data toward a laboratory diagnosis of syphilis. Indeed, a number of studies have described nucleic acid amplification tests for the detection of *T. pallidum*-specific nucleic acid from genital swab specimens collected from both genders (2–4).

As it pertains to MSM, non-invasive rectal swab screening has been demonstrated in numerous venues to be an effective means of detecting sexually transmitted infection (STI) agents (5–7). Specific investigations into diagnostic yield from self-collected rectal swabs have revealed high levels of concordance with provider collection (8, 9). In a preliminary study, Zapp et al. (10) performed a serologic correlation of a transcription-mediated amplification (TMA)-based research-use-only (RUO) assay for detection of *T. pallidum* 23S ribosomal (r)RNA from self-collected rectal swab specimens of an MSM cohort, with all *T. pallidum*-detected specimens coinciding with reactive non-treponemal results.

Because the inherent nature of this anatomic site collection may be fraught with abundances of microbiota (including normal flora treponemes [11]), cross-reacting substances, interfering substances, potentially autofluorescence substances, and nucleic acid amplification inhibitors, we sought to perform additional *ex vivo* challenges of this RUO assay to confirm test specificity. Moreover, experiments were undertaken to fully elucidate assay sensitivity.

## MATERIALS AND METHODS

### RUO *T. pallidum* TMA assay

Proprietary reagents were supplied by Hologic, Inc. (San Diego, CA). A total of 50 μL volume of target capture oligonucleotide, T7 initiator oligonucleotide, T7- and non-T7-based primer oligonucleotides specific to 23S rRNA sequences of *T. pallidum*, and probe oligonucleotide were added by the end-user into general-purpose target capture, amplification, enzyme, or FAM fluorescence-based promoter reagents (12). Automated direct-tube sample processing of the RUO assay occurred on the Panther system utilizing real-time TMA in tandem with fluorescence emission detection (13). System software recorded assay-specific FAM fluorescence values at specific intervals throughout the assay. Analyzer-programmed parameters for a positive result included a FAM fluorescence signal emergence time (T-time) cutoff value of 40 minutes and relative fluorescent unit output exceeding 1,000 units.

### Rectal swab specimens

The study utilized remnant rectal swab specimens that were collected using the Aptima Multitest Swab Specimen Collection Kit (Hologic) as part of routine clinical testing and research studies. The current investigation was approved by the Marquette University Institutional Review Board. Rectal swabs were previously submitted for routine performance of a *Chlamydia trachomatis*- and *Neisseria gonorrhoeae*-specific commercial FDA-cleared TMA assay, as well as a laboratory-verified *Mycoplasma genitalium* TMA assay (6, 7). All specimens were maintained at 2°C–30°C prior to testing. All specimens were tested within 30 days of collection.

### Rectal swab matrix

Residual rectal swab specimen transport medium from specimens that failed to generate significant T-time and qualitative result data, upon performance of RUO *T. pallidum* TMA, was pooled into a 50 mL conical vial and utilized as a matrix for subsequent experimentation relative to interfering/inhibitory substances and for limit of detection studies.

### RNA extraction from non-*T*. *pallidum* treponemal bacteria

RNA was extracted from *Treponema denticola*, *Treponema phagedenis*, and *Treponema refringens* using the PureLink RNA minikit (Life Technologies, Carlsbad, CA). RNA concentrations were estimated with the Trizol-Qubit method using a Qubit RNA HS/BR Assay Kit (ThermoFisher Scientific, Waltham, MA) following the manufacturer’s instructions.

### *T. pallidum* whole organism and *in vitro* RNA transcript material

Control materials used in these experiments included synthetic *in vitro* RNA transcripts corresponding to region 1 of *T. pallidum* 23S rRNA, which were diluted in Aptima specimen transport medium (Hologic). Whole-organism *T. pallidum* (Nichols strain) and *Treponema endemicum* were propagated in rabbit testes, quantitated by darkfield microscopy, and diluted in Aptima specimen transport medium.

### Inhibitory/interfering substances

In a first set of experiments, one swab tip-full of commercial gynecologic lubricant was added to the bottom of clean, conical-bottom specimen tubes, followed by 450 μL aliquots of rectal swab matrix. In analogous fashion, one small spatula tip-full of commercial talcum powder was added to the bottom of clean, conical bottom specimen tubes, followed by 450 μL aliquots of rectal swab matrix. A subset of tubes within each treatment group was spiked with a 100 μL aliquot of *T. pallidum* Nichols strain organism suspension at a concentration of 10^4^/mL. In a second set of experiments, human whole blood, seminal fluid, or urine was dispensed into the bottom of clean, conical bottom specimen tubes, followed by appropriate volumes of rectal swab matrix to create ratios of 1% whole blood, 10% seminal fluid, and 20% urine. Total volumes devised in these experiments were 3,000 μL. A subset of tubes within each treatment group was spiked with a 300 μL aliquot of *T. pallidum* Nichols strain organism suspension at a concentration of 10^4^/mL. All tubes were subjected to RUO *T. pallidum* TMA and processed by Panther direct tube sampling.

In a third set of experiments, non-*T*. *pallidum Treponema* spp. RNA was added to Aptima Multitest Swab Specimen Collection tubes containing 2.9 mL of Aptima sample transport medium (Hologic) for RUO *T. pallidum* TMA testing by direct tube sampling on the Panther system. Experimentation was undertaken with multiple volume deliveries; maximum RNA concentrations tested were 2,788 ng/mL for *T. denticola*, 248.0 ng/mL for *T. phagedenis*, and 643.6 ng/mL for *T. refringens*. With respect to all experimentation described in this section, generated data were compared to respective control tubes containing solely rectal swab matrix or Aptima specimen transport medium.

### Limit of detection experiments

*T. pallidum* whole organism stocks and *in vitro* RNA transcript solutions were serially diluted 10-fold in separate diluents of Aptima specimen transport medium and rectal swab matrix. All samples were subjected to RUO *T. pallidum* TMA and processed by Panther direct tube sampling. Generated data were compared to respective control tubes containing solely Aptima specimen transport medium or rectal swab matrix.

### Statistical analysis

Differences in mean endpoint FAM fluorescence were analyzed by the *t*-test for independent samples. Positivity results were analyzed by probit analysis (normal model) to determine the 50% and 95% limit of detection values. An *a priori* decision was made to examine statistical significance using a two-tailed test with an alpha level of 0.05.

## RESULTS

### Specificity and cross-reactivity assessment of RUO *T. pallidum* TMA

Analytical specificity of RUO *T. pallidum* TMA was first assessed by testing specimen transport medium in the absence or presence of various concentrations of *T. denticola*-, *T. phagedenis*-, and *T. refringens*-specific RNA (Table 1). None of the specimen transport medium tubes spiked with RNA samples demonstrated false-positive results for RUO *T. pallidum* TMA. These non-*T*. *pallidum Treponema* spp. exhibited mean endpoint FAM fluorescence values ranging from 620.7 to 633.5 units (*P* ≥ 0.22 versus specimen transport medium; Table 1). As part of an analyzer interpretation algorithm, endpoint FAM fluorescence values exceeding 1,000 units contribute to a positive result for RUO *T. pallidum* TMA.

**TABLE 1:** Performance of RUO *Treponema pallidum* TMA on specimen transport medium with additive non-*T*. *pallidum Treponema* spp. RNA

Non-*Treponema pallidum* *Treponema* spp. added	Number of assessments	Range of RNA concentration added (ng/mL)	Mean endpoint FAM fluorescence (95% CI)
*Treponema denticola*	22	65.3–2,788.7	620.7 (589.4, 652.0)
*Treponema phagedenis*	22	1.6–248.0	632.3 (599.7, 664.9)
*Treponema refringens*	22	25.4–643.6	633.5 (603.9, 663.1)
[Specimen transport medium]	43		639.8 (624.0, 655.6)

In ancillary experimentation, challenge of RUO *T. pallidum* TMA with *T. endemicum* at a concentration of 10^1^ cells/mL produced significant FAM fluorescence. Thirteen such replicates yielded a mean endpoint FAM fluorescence value of 3,949.9 units (95% CI 3,182.7, 4,717.1; *P* < 0.0002 versus specimen transport medium).

Secondly, the introduction of exogenous and endogenous substances to the rectal swab matrix was performed, in part, to determine if the substances generate autofluorescence. In a point prevalence study, 3,586 rectal swab specimens exhibited a mean endpoint FAM fluorescence value of 637.5 units (95% CI 635.2–639.8) by RUO *T. pallidum* TMA. Addition of commercial talcum powder, gynecologic lubricant, 1% blood, 10% seminal fluid, or 20% urine to pooled rectal swab matrix did not result in increased mean endpoint FAM fluorescence (mean values of individual substances ranged from 624.0 to 654.4 units) as compared to control rectal swab matrix (*P* ≥ 0.20; Table 2). Moreover, the addition of these exogenous and endogenous substances to suspensions of the *T. pallidum* Nichols strain resulted in exponential FAM fluorescence kinetic profiles that were similar to those from amplification of *T. pallidum* alone. Specifically, spiking *T. pallidum* Nichols strain to tubes containing powder and lubricant (i.e., non-liquid exogenous substances) resulted in mean endpoint FAM fluorescence values of 4,190.6 and 4,239.6 units, respectively (*P* ≥ 0.68; Table 3), as tested by RUO *T. pallidum* TMA. Analogous addition of liquid-based endogenous substances (blood, seminal fluid, urine) resulted in mean endpoint FAM fluorescence values that were similar to those of *T. pallidum* rRNA amplification alone (*P* ≥ 0.14).

**TABLE 2:** Performance of RUO *Treponema pallidum* TMA on rectal swab matrix with additive exogenous or endogenous substances

Additive exogenous/endogenous substance	Number of assessments	Mean endpoint FAM fluorescence (95% CI)
Commercial talcum powder	84	654.4 (622.4, 686.4)
Lubricant	83	628.3 (612.0, 644.6)
Blood (1% vol/vol)	98	631.4 (616.9, 645.9)
Seminal fluid (10% vol/vol)	98	624.0 (605.8, 642.2)
Urine (20% vol/vol)	98	629.0 (613.5, 644.5)
[Rectal swab matrix]	98	633.0 (618.7, 647.3)

**TABLE 3:** Performance of RUO *Treponema pallidum* TMA on suspensions of *Treponema pallidum* Nichols strain with additive exogenous or endogenous substances

Number of *T. pallidum* Nichols strain assessments	Mean endpoint FAM fluorescence of *T. pallidum* Nichols strain (95% CI)	Additive exogenous/endogenous substance	Number of *T. pallidum* Nichols strain assessments in the presence of an additive	Mean endpoint FAM fluorescence of *T. pallidum* Nichols strain in the presence of an additive (95% CI)	*P* value versus control
68	4,258.3 (3,977.4, 4,539.2)	Commercial talcum powder	84	4,190.6 (3,995.6, 4,385.6)	0.68
		Lubricant	84	4,239.6 (4,066.5, 4,412.7)	0.90
62	4,020.0 (3,773.3, 4,266.7)	1% (vol/vol) blood	90	4,237.1 (4,063.0, 4,411.2)	0.14
		10% (vol/vol) seminal fluid	90	3,850.7 (3,632.0, 4,069.4)	0.31
		20% (vol/vol) urine	90	3,971.4 (3,772.3, 4,170.5)	0.76

### RUO *T. pallidum* TMA limit of detection

Further characterization of RUO *T. pallidum* TMA entailed serial 10-fold dilutions of *in vitro* transcript and whole cell *T. pallidum* in mock and clinical matrices. For titrations in pooled rectal swab matrix, positive results were reported in all replicates tested at concentrations ≥1,000 copies/mL of *in vitro* 23S rRNA transcript (*n* = 30 replicates for each of three concentrations tested) and in >97% of additional replicates tested at concentrations ≥10 *T*. *pallidum* cells/mL (*n* ≥ 35 replicates for two concentrations tested). For titrations in specimen transport medium, positive results were reported in >96% of replicates tested at concentrations ≥10,000 copies/mL of *in vitro* 23S rRNA transcript (*n* = 30 replicates for each of two concentrations tested) and in 70% of replicates tested at a concentration of 1,000 copies/mL. Relative to rectal swab matrix, T-times were observed in >82% of replicates tested at concentrations ≥10 *T*. *pallidum* cells/mL (*n* ≥ 32 replicates for each of two concentrations tested) and in 8.6% of replicates tested at a concentration of 1 T. *pallidum* cell/mL (35 replicates tested).

Via probit analysis, these data translated into limits of detection that were found to be lower within the pooled rectal swab matrix (Table 4). As one example, the 95% limit of detection value for *ex vivo* detections of whole cell *T. pallidum* in rectal swab matrix was 9 cells/mL as compared to 48 cells/mL in specimen transport medium. In a similar fashion, 95% limit of detection values for *in vitro* challenges of 23S rRNA were 421 copies/mL and 5,707 copies/mL in rectal swab and specimen transport matrices, respectively.

**TABLE 4:** Probit analysis of serial 10-fold dilution experiments using *Treponema pallidum* whole cell and *in vitro* 23S rRNA transcripts as challenges to determine RUO *T. pallidum* TMA limit of detection

Matrix	Challenge	Limit of detection value (%)	Result (lower limit, upper limit)	McFadden R^2^ coefficient
Specimen transport medium	*T. pallidum* whole cell	50	5 (3–8) cells/mL	0.911
		95	48 (24–143) cells/mL	0.911
	*T. pallidum* 23S rRNA *in vitro* transcript	50	221 (124–396) copies/mL	0.927
		95	5,707 (2,454–21,111) copies/mL	0.927
Rectal swab matrix	*T. pallidum* whole cell	50	3 (2, 5) cells/mL	0.985
		95	9 (6, 16) cells/mL	0.985
	*T. pallidum* 23S rRNA *in vitro* transcript	50	316 (none given) copies/mL	1
		95	421 (none given) copies/mL	1

## DISCUSSION

Early studies of RUO *T. pallidum* TMA sought to integrate organism detection from MSM patients with syphilis serology and clinical presentation. Golden et al. (14) performed the assay on both rectal and pharyngeal mucosal swab specimens and reported that within 24 patients who met study-defined criteria for diagnosis of syphilis, one-half had a positive result from either site of collection. Within these 12 patients, there were two who tested positive by the assay in the absence of both a reactive RPR result and a clinical diagnosis of syphilis. Zapp et al. (10) corroborated a portion of these findings in an independent MSM cohort by demonstrating positive RUO *T. pallidum* TMA results from rectal swab specimens of five participants with non-treponemal serologic seroconversion. In one additional patient, RUO *T. pallidum* TMA detection was accompanied by an RPR titer that subsequently showed a fourfold reduction after 120 days.

The aforementioned clinical data imply that RUO *T. pallidum* TMA possesses robust analytic sensitivity. This paradigm is not without precedent, as other publications have noted increased sensitivity of TMA-based assays over those of other nucleic acid amplification modalities specific to *C. trachomatis* (15), *N. gonorrhoeae* (16), *Trichomonas vaginalis* (17), and *M. genitalium* (18). Improved performance in a clinical setting has also been documented with hepatitis C virus TMA (19). In our experimentation, the introduction of potentially inhibitory substances such as urine, lubricant, and blood to specimen transport medium tubes containing known concentrations of *T. pallidum* Nichols strain (Table 3) did not result in a significant reduction of mean endpoint FAM fluorescence. The prospect of a concomitant lower limit of detection for TMA was confirmed by probit analysis (95% parameter; Table 4), which revealed *T. pallidum* whole organism detection at a range of 9–48 cells, with one set of determinations made in a clinical matrix. These data hold particular significance when considering the biology of *T. pallidum* infection. Mahoney and Bryant (20) reported that *T. pallidum* applied to mucous membranes could be localized to deep tissue within 2 hours. In attempts to detect the agent of syphilis by non-serologic means (i.e., mucocutaneous direct detection), one must be cognizant of potentially low-level residual organisms present at the original site of acquisition. TMA-based diagnostics may be indicated for such scenarios. Additional studies will be necessary to determine the potential utility of RUO *T. pallidum* TMA in early screening of asymptomatic (both female and male) patient populations who may be at increased risk for *T. pallidum* infection.

Potential increased assay analytic sensitivity, by way of a fluorescence-based detection system, could be explained by potential cross-reactivity and interfering substances. To test this hypothesis, specimen transport medium tubes were mock challenged with nucleic acids from three non-*T*. *pallidum Treponema* spp. that could simulate inhabitants of mucocutaneous tissues in humans. Mean endpoint FAM fluorescence values (none of which exceeded 634 units; Table 1) were not significantly altered from those derived from analysis of control material (*P* ≥ 0.22). Endpoint FAM fluorescence values exceeding 1,000 units contribute to a RUO *T. pallidum* TMA result of positive. One non-*T*. *pallidum Treponema* spp. that did not follow this RUO *T. pallidum* TMA specificity paradigm was the endemic treponematosis agent *T. endemicum*. However, these findings were not surprising since agents of endemic treponematoses have been demonstrated to be >99.7% identical to *T. pallidum* at the genetic level (21). Moreover, clinical laboratorians are cognizant of the fact that *T. endemicum*, the etiologic agent of endemic syphilis (bejel), has a typical distribution in dry, hot nations, with major localization in the Sahelian region of Africa (22).

With respect to the possibility of non-specific fluorescence or autofluorescence generation in the context of our study, Bottiroli et al. (23) reported natural fluorescence *ex vivo* in both normal and neoplastic human colonic tissue. However, data presented in Table 2 revealed a stable mean endpoint FAM fluorescence generated by RUO *T. pallidum* TMA in 98 assessments of pooled rectal swab matrix; moreover, addition of several exogenous or endogenous substances (including powder and seminal fluid) to known rectal swab matrices not exhibiting FAM fluorescence did not generate non-specific FAM fluorescence (*P* ≥ 0.20).

The interpretation of findings presented in this report is not without limitations. One major limitation relates to potential variability in run-to-run fluorescence output, even in background matrix material. This potential is hypothetically amplified by the fact that the RUO *T. pallidum* TMA is constructed in 100-test kits, meaning that multiple user-constructed kits were necessary to complete work in this investigation. This potential variability relative to kit construction is inherent to other commercially available TMA assays and may actually be generalizable to routine performance of the assay. Furthermore, larger *n* values for treatment groups executed in this study would mitigate variability to a degree for valid statistical analysis. Finally, data from the talcum powder and lubricant experiments may not extrapolate to similar products that have different chemical compositions.

In conclusion, RUO *T. pallidum* TMA has the potential to improve laboratory diagnosis of syphilis, particularly in patients at increased risk for STIs. The high analytic sensitivity and lack of cross-reactivity of the RUO *T. pallidum* TMA can facilitate other laboratories exploring the use of the assay in a research setting. Further clinical correlation in MSM at increased STI risk, as well as from female genital specimens (24), is warranted.
